# SMTP‐44D improves diabetic neuropathy symptoms in mice through its antioxidant and anti‐inflammatory activities

**DOI:** 10.1002/prp2.648

**Published:** 2020-11-20

**Authors:** Ryosuke Shinouchi, Keita Shibata, Terumasa Hashimoto, Shiori Jono, Keiji Hasumi, Koji Nobe

**Affiliations:** ^1^ Division of Pharmacology Department of Pharmacology, Toxicology & Therapeutics School of Pharmacy Showa University Shinagawa‐ku Tokyo Japan; ^2^ Pharmacology Research Center Showa University Shinagawa‐ku Tokyo Japan; ^3^ Department of Applied Biological Science Tokyo University of Agriculture and Technology Fuchu‐shi Tokyo Japan; ^4^ TMS Co., Ltd Fuchu‐shi Tokyo Japan

**Keywords:** allodynia, complication, diabetic neuropathy, hyperalgesia, inflammation, oxidative stress

## Abstract

Diabetic neuropathy (DN) is one of the major complications of diabetes. However, there are few approved effective therapies for painful or insensate DN. Recent studies have implicated oxidative stress and inflammation in the pathogenesis of DN, and suppressing these could be an important therapeutic strategy. We previously reported that *Stachybotrys microspora* triprenyl phenol‐44D (SMTP‐44D) exhibits both antioxidant and anti‐inflammatory activities. The aim of this study was to evaluate the effects of SMTP‐44D in a mouse model of streptozotocin‐induced DN. SMTP‐44D was administered for 3 weeks after the disease induction, and its effects were evaluated on the basis of mechanical and thermal thresholds, blood flow in the bilateral hind paw, and blood flow and conduction velocity in the sciatic nerve. Furthermore, the levels of inflammatory factors, such as tumor necrosis factor (TNF)‐α, interleukin (IL)‐1β, IL‐6 and malondialdehyde (MDA), in the sciatic nerve were assessed. Neurological degeneration was assessed by measuring myelin thickness and g‐ratio in the sciatic nerve. SMTP‐44D treatment significantly improved allodynia, hyperalgesia, blood flow, and conduction velocity in DN model mice in a dose‐dependent manner. Neurological degeneration was also significantly improved, accompanied by decreased levels of inflammatory factors (TNF‐α, 57.8%; IL‐1β, 51.4%; IL‐6, 62.8%; and MDA, 40.7% reduction rate against the diabetes mellitus + normal saline group). Thus, SMTP‐44D can improve allodynia and hyperalgesia in DN without affecting the body weight and blood glucose levels, which may be due to its antioxidant and anti‐inflammatory properties. In conclusion, SMTP‐44D could be a potential therapeutic agent for the treatment of DN.

**Abbreviations**
BCA: bicinchoninic acid
DM: diabetic mellitus
DN: diabetic neuropathy
EDR: edaravone
ELISA: enzyme‐linked immunosorbent assay
IL: interleukin
i.p.: intraperitoneal
MDA: malondialdehyde
OH: hydroxyl radicals
PGN: pregabalin
PIC: protease inhibitor cocktail
PU: perfusion unit
ROS: reactive oxygen species
SMTP‐44D: *stachybotrys microspora* triprenyl phenol‐44D
STZ: streptozotocin
TBARS: thiobarbituric acid reactive substances
TNF: tumor necrosis factor

**Significance statement**

This study shows that SMTP‐44D can improve the neural function, mechanical allodynia, and thermal hyperalgesia caused by diabetic neuropathy and these effects were accompanied by the reduction of lipid peroxidation, and improve the levels of tumor necrosis factor‐α, interleukin‐1β, and interleukin‐6. This novel finding suggests that SMTP‐44D has therapeutic potential against diabetic neuropathy.

## 1 INTRODUCTION

A worldwide prevalence of 425 million people with diabetes was estimated in 2017 and it is expected to increase to 693 million people in 2045.[1] Lifelong diabetes treatment incurs massive medical, economic, and social burden. Diabetic neuropathy (DN), which is one of the major complications of diabetic mellitus, affects more than 50% of the diabetic patients.[2] Regarding the pathologic progression of DN, the sensory defects, such as allodynia and hyperalgesia, occur at the early stages of this pathological condition. Sensory paralysis involving nerve loss and degeneration of axons occur at the advanced stages, and often lead to foot amputation due to diabetic foot ulcers.[3, 4] Thus, DN is a social problem that remarkably reduces the quality of life and causes massive increase in the medical costs. Thus, researchers have focused efforts toward the development of new therapeutic targets against DN.

In general, the persistence of hyperglycemia activates the protein kinase C pathway, polyol pathway, advanced glycation end products pathway, and hexosamine pathway. Oxidative stress is involved in the activation of all these pathways[5, 6] and causes sensory defects, a decrease in blood flow, a delay in conduction velocity, and the degeneration of Schwann cells.[7, 8] Furthermore, recent studies have reported that not only oxidative stress but also inflammation plays an important role in tissue damage in diabetes.[9, 10, 11] The upregulation of inflammatory cytokines has been reported in various types of diabetes and plays a vital role in the structural and functional damage of the peripheral nerves, leading to DN.[12, 13, 14] Oxidative stress and inflammation have been shown to be involved in the pathogenesis of DN, and these are considered important therapeutic targets.[15] Currently, aldose reductase inhibitors, ubiquinone (Coenzyme Q10, vitamin B12, and tricyclic antidepressants are used as the therapeutic agents for DN. However, they only provide symptomatic relief,there are very few approved effective therapies for painful or insensate DN.[16, 17, 18] The underlying etiology of DN is multifactorial, and multiple pathways are associated with its onset. However, the detailed mechanism underlying DN has not been clarified yet.

*Stachybotrys microspora* triprenyl phenols (SMTPs) are a family of small molecule triprenyl phenol metabolites that are derived from the fungus *S microspora*.[19] Among the SMTPs, SMTP‐44D was reported to have effective antioxidant and anti‐inflammatory activities.[20, 21, 22] However, it remains unknown whether SMTP‐44D has therapeutic effects in DN or if its antioxidant and anti‐inflammatory activities could improve allodynia, hyperalgesia, decrease in blood flow, delay of conduction velocity, and neurological degeneration in DN.

Thus, the present study aimed to evaluate the effect of SMTP‐44D on DN, and to elucidate the underlying mechanism involved in relation to its activity on oxidative stress and inflammation.

## 2 MATERIALS AND METHODS

### 2.1 Chemicals and drugs

**Figure 1:**
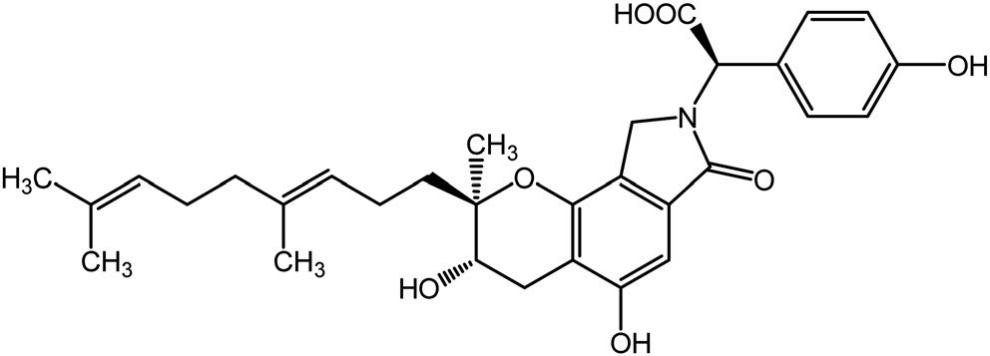
Chemical structure of SMTP‐44D

Streptozotocin (STZ) and protease inhibitor cocktail (PIC) were purchased from Sigma‐Aldrich Co., LLC. (Missouri, USA). Isoflurane, 10% formalin neutral buffer solution (pH 7.4), and liquid paraffin were purchased from FUJIFILM Wako Pure Chemical Corporation (Osaka, Japan). Hair‐removing body cream was purchased from Kracie Holdings., Ltd. (Tokyo, Japan). RIPA buffer was purchased from Cayman Chemical Company (Michigan, USA). Edaravone (Radicut®) was purchased from Mitsubishi Tanabe Pharma Corporation (Osaka, Japan). Pregabalin (Lyrica®) was purchased from Pfizer Inc (Tokyo, Japan). SMTP‐44D was generously donated by TMS Co., Ltd. (Tokyo, Japan). The structure of SMTP‐44D is shown in Figure 1.

### 2.2 Animals

Nine‐week old C57BL/6J male mice were purchased from CLEA Japan, Inc (Tokyo, Japan). The mice were habituated to laboratory conditions for 1 week before the study. The animals were maintained in an air‐conditioned animal room at 23 ± 2°C, with 50 ± 20% relative humidity and under 12‐hour light‐dark cycle conditions (light on from 8 am to 8 pm). The animals had access to food and water available ad libitum except during the experiment. The mice were randomly selected, and the experiments were performed without blinding. All experiments were conducted in accordance with the regulations of the Committee of Animal Care and Welfare of the Showa University (permit number: 29008) and the Guide for the Care and Use of Laboratory Animals as adopted and promulgated by the US National Institutes of Health.

### 2.3 Development of the mouse model of diabetic neuropathy

Diabetes was induced by a single intraperitoneal (i.p.) injection of STZ (200 mg/kg). The STZ i.p. injection was immediately carried out by dissolving it in normal saline (NS). 7 days after the STZ i.p. injection, the diabetes induction was confirmed by determining the blood glucose levels in the blood sample withdrawn from the tail vein using the self‐testing glucose meter (Medisafe mini GR‐102; Terumo Corporation, Tokyo, Japan). The mice with blood glucose levels ≥400 mg/dL were considered diabetic and were selected for the experiments. The nondiabetic mice were injected with an equal volume of NS.

### 2.4 Treatment schedule

The von Frey test, hot plate test, and the measurements of the body weight, blood flow in bilateral hind paw, and blood glucose levels were conducted before, and 7, 14, 21, and 28 days after the STZ treatment. Furthermore, 28 days after the STZ treatment, the blood flow, conduction velocity, oxidative stress, and inflammatory cytokine levels in the sciatic nerve were measured. In addition, the myelin thickness and g‐ratio in the sciatic nerve were measured. SMTP‐44D (0.3, 3, and 30 mg/kg/day, i.p.), edaravone (EDR, 10 mg/kg/day, i.p.), pregabalin (PGN, 10 mg/kg/day, i.p.), and NS (i.p.) were administered to the diabetic mice for 21 consecutive days, starting 8 days after the STZ injection. The nondiabetic mice were administered NS. All treatments were injected intraperitoneally in a volume of 0.25 mL/10 g body weight.

### 2.5 Assessment of mechanical allodynia by the Von Frey test

The mechanical thresholds were measured using the Dynamic Planter Aesthesiometer (Ugo Basile Biological Research Apparatus Company, Varese, Italy). The von Frey test was performed according to the previous report with some modifications.[23] In brief, mice were placed in clear acrylic boxes (10 cm × 10 cm × 14 cm) with metal grid floors in a temperature‐ and humidity‐controlled room (23 ± 2°C and 50 ± 20%, respectively), and were habituated for 1 hour before the von Frey test. A stimulus was applied via a metal filament (0.5 mm in diameter) that applied a linearly increasing force ramp (1 g/s) ranging up to 10 g in 10 seconds to the plantar surface of the hind paw. The hind paw withdrawal threshold was calculated as the average of three consecutive tests with at least 3 minutes interval between each trial.

### 2.6 Assessment of thermal hyperalgesia by the hot plate test

The thermal thresholds were measured using EC‐1200 Hot Plate (Iuchi Corporation, Osaka, Japan). The hot plate test was performed according to the previous report with some modifications.[24] In brief, 30 minutes before the hot plate test, the mice were acclimated as mentioned above for the von Frey test. The mice were placed into a Plexiglas cylinder (diameter, 20 cm, height, 25 cm) on the heated plate maintained at 49‐50°C. The latency was recorded as the time between keeping the mouse onto the plate until the pain reaction, as indicated by the jumping or hind paw licking. The latency time was calculated as the average of three consecutive tests performed at 30 minutes intervals. The cut‐off time was 30 seconds to prevent tissue damage and if the mice did not respond to pain reaction within 30 seconds, it was recorded as 30 seconds.

### 2.7 Measurement of blood flow in the bilateral hind paw skin

The blood flow in the bilateral hind paw skin was measured using a laser Doppler flowmeter (Moor Instruments Ltd., Devon, United Kingdom). The mice were anesthetized by isoflurane (initiation, 5%; maintenance, 2%). The evaluation was immediately conducted to minimize the effect on body temperature. The measurement was repeated twice in the same region of interest (ROI). The laser Doppler signal of the scanning image was analyzed using the MoorFLPI software (version 2.1). This software enabled the analysis of perfusion values within a ROI, in areas of the same size containing the same number of pixels. The blood flow in the bilateral hind paw skin was as calculated as the average of right and left of arbitrary perfusion units.

### 2.8 Measurement of blood flow in the sciatic nerve

Blood flow in the bilateral sciatic nerve 28 days after the STZ treatment was measured using a laser Doppler flowmeter (Moor Instruments Ltd., Devon, UK). The mice were anesthetized by isoflurane (initiation, 5%; maintenance, 2%), and the fur from the back to the hind limbs was completely removed using an electric shaver and hair‐removing body cream. To remove any residual hair‐removing body cream, the skin was cleaned carefully with an alcohol wipe and dry wipe. To minimize the reduction of body temperature, the skin was maintained at 37°C using HOT‐1 (ALA Scientific Instruments Inc, New York, NY, USA) and HEATINGPAD‐1 (ALA Scientific Instruments Inc), and the entire procedure of blood flow and conduction velocity was completed within 1 hour. The skin was incised from the quadriceps muscle to the gastrocnemius muscle in right hind paw, and the sciatic nerve was exposed. Subsequently, this tissue was covered with liquid paraffin to avoid tissue dehydration. The measurement of blood flow in the sciatic nerve was performed using a procedure similar to the measurement of the blood flow in the bilateral hind paw skin. To unify the recording time, blood flow in the right sciatic nerve was measured 20 minutes and left sciatic nerve was measured 40 minutes after anesthesia. The blood flow in the sciatic nerve was calculated as the average of right and left of arbitrary perfusion units. The measurement of blood flow in the left sciatic nerve was performed after the measurement of conduction velocity in the right sciatic nerve.

### 2.9 Measurement of conduction velocity in the sciatic nerve

After the measurement of blood flow in the right sciatic nerve, the conduction velocity in the right sciatic nerve was measured using PowerLab 4/26 (AD Instruments Ltd., New South Wales, Australia), Analog Stimulus Isolater model 2200 (A‐M Systems Inc, Washington, USA), and ER‐1 Extracellular Amplifier (Cygnus Technology Inc, Pennsylvania, USA). The conduction velocity was measured according to the previous report with some modifications.[25, 26] For the measurement of the conduction velocity in the sciatic nerve, Vernier caliper was used to measure the distances between the proximal and the distal sciatic nerve. Bipolar needle electrodes with a width of 1 mm and silver ring electrodes were used for the stimulation and recording, respectively. The silver ring electrodes and ground electrodes were placed on the gastrocnemius muscle. The sciatic nerve stimulation induced by bipolar needle electrodes was recorded with single square wave pulse (5 V in intensity, 50 μs in width, and 0.1 mA/V) using PowerLab Stimulus Isolator and Extracellular Amplifier. Records measured by single square wave pulse were analyzed for conduction velocity using LabChart (version 7.3.8). This application enabled the analysis of the latency of the signal response and the duration and amplitude of the signal. The action potential latency (L) of the sciatic nerve and the distance (D) of stimulation between the proximal and distal sciatic nerves were measured to calculate the conduction velocity in the sciatic nerve (D/L = m/s). To produce reliable data, the recordings were done 10 times independently for the optimal response curves per stimulation site, and their average of right and left was calculated. The measurement of conduction velocity in the left sciatic nerve was performed after the measurement of blood flow in the left sciatic nerve.

### 2.10 Morphological assessment

The sciatic nerve was dissected and fixed by 10% formalin neutral buffer solution. After Epon resin embedding, a semi‐thin section was prepared and stained with toluidine blue. The digitized images were acquired at 400‐fold magnification on a microscope (AX; Olympus Corporation., Tokyo, Japan) equipped with a digital camera (E‐330; Olympus Corporation., Tokyo, Japan). For the quantitation of myelin thickness and g‐ratio analyses, ImageJ software version 1.52a (National Institutes of Health, Maryland, USA) was used to determine the axon diameter and the myelinated fiber diameter (axon + myelin). The myelin thickness was calculated as follow:

$$\text{myelin thickness}=\left(\text{myelinated fiber diameter}‐\text{axon diameter}\right)/2.$$

The g‐ratio was calculated as follow:

g‐ratio=axon diameter/myelinated fiber diameter.

The myelin thickness and g‐ratio were evaluated for at least 200 randomly selected fibers per mouse.

### 2.11 Tissue sampling

At the 28 days after the STZ treatment, the mice were euthanized and both of the sciatic nerves were removed, washed with ice‐cold phosphate‐buffered saline solution (137 mM NaCl, 8.1 mM Na_2_HPO_4_, 2.7 mM KCl, and 1.47 mM KH_2_PO_4_), frozen immediately in liquid nitrogen, and stored at −80°C until they were examined for the levels of oxidative stress and inflammatory cytokines.

### 2.12 Determination of the levels of oxidative stress

The levels of oxidative stress were determined by assessing the extent of lipid peroxidation by measuring the levels of malondialdehyde (MDA) using the thiobarbituric acid reactive substances (TBARS) assay kit (Cayman Chemical, Michigan, USA). The levels of total protein were quantitated by using the Pierce^TM^ bicinchoninic acid (BCA) protein assay kit (Thermo Fisher Scientific Inc, Minnesota, USA) according to the manufacturer's manual. In brief, the frozen sciatic nerve was homogenized in 250 µL of RIPA buffer with PIC and centrifuged at 1600 *g* for 15 minutes at 4°C; the supernatant was collected and used for the measurement of the levels of MDA and total protein. For measurement of MDA levels, the supernatant was stimulated by the addition of sodium dodecyl sulfate solution. The reactions were terminated by adding acetic acid solution and thiobarbituric acid reactive substance reagent. After boiling for 1 hour, the samples were cooled on ice for 30 minutes and centrifuged at 1600 *g* for 10 minutes at 4°C. The samples were collected and were used to measure the MDA levels at 535 nm using Spectra Max i3 spectrophotometer (Molecular Devices LLC., Tokyo, Japan). The levels of MDA were determined using the standard curve and were calculated as nmol/mg total protein content.

### 2.13 Determination of the levels of inflammatory cytokines in sciatic nerve

The levels of inflammatory cytokines, tumor necrosis factor (TNF)‐α, interleukin (IL)‐1β, and IL‐6, were assessed using the Quantikine enzyme‐linked immunosorbent assay (ELISA) kit (R&D Systems Inc, Minnesota, USA) according to the manufacturer's manual. In brief, the frozen sciatic nerve samples were homogenized in 250 µL of RIPA buffer with PIC and centrifuged at 10 000 *g* for 15 minutes at 4°C; the supernatant was collected and used for the measurement of the levels of TNF‐α, IL‐1β, IL‐6, and total protein. The levels of these cytokines were determined by interpolation from the standard curves and normalized for protein content for each sample, and are expressed as pg/mg total protein content.

### 2.14 Statistics

All data were expressed as the mean ± SD and checked for homogeneity of variance using the Shapiro‐Wilk test. If the variance was homogeneous, the data were evaluated using two‐way repeated measures ANOVA followed by Dunn's comparison test or one‐way ANOVA followed by Bonferroni test. If not, Kruskal‐Wallis ANOVA was applied, followed by the Dunn's comparison test. Two‐way repeated measures ANOVA followed by the Dunn's comparison test was used to compare diabetic mice with nondiabetic mice in order to properly evaluate the pharmacological effects of the treatments over time (0, 7, 14, 21 and 28 days) of the von Frey test, hot plate test, and the measurements of the body weight, blood flow in bilateral hind paw, and blood glucose levels. Moreover, comparisons between diabetic and treatment mice at 14, 21 and 28 days were measured by one‐way ANOVA followed by Bonferroni test. *P* < .05 was considered statistically significant.

## 3 RESULTS

### 3.1 Changes in body weight and blood glucose levels following the administration of SMTP‐44D in STZ‐induced diabetic mice

**Figure 2:**
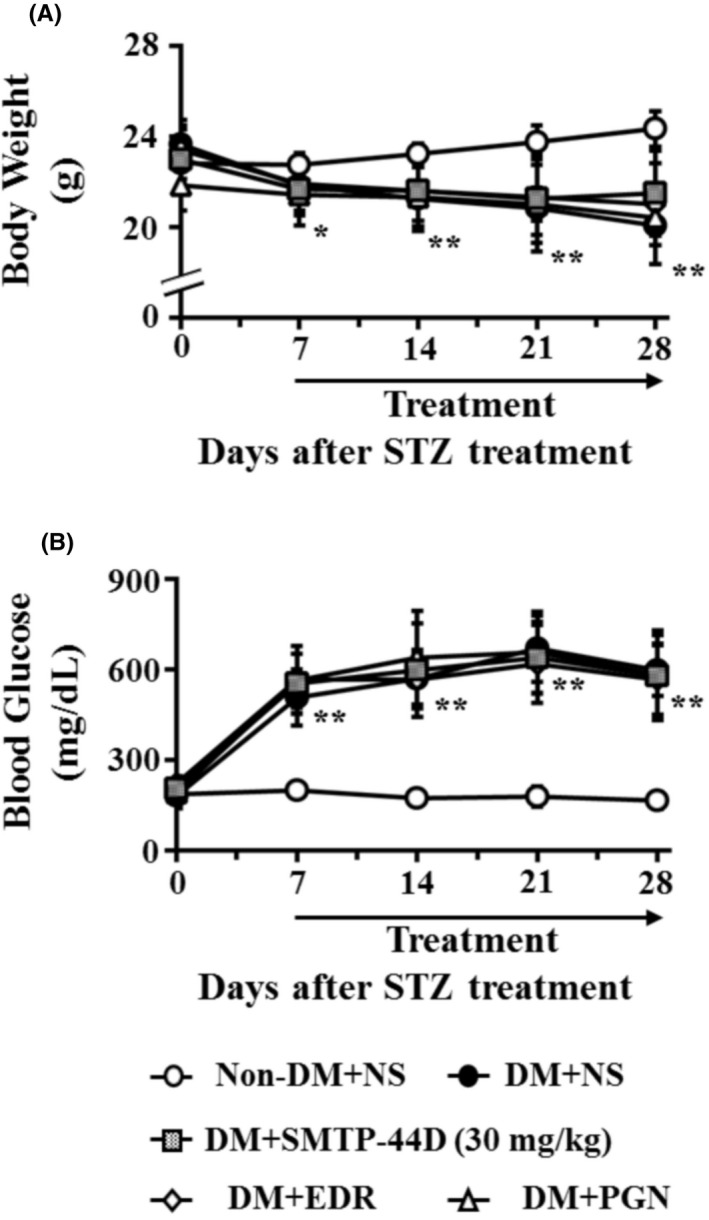
Time course of changes in body weight (A) and blood glucose levels (B) in mouse model of DN. SMTP‐44D (30 mg/kg, intraperitoneally [i.p.]), EDR (10 mg/kg, i.p.), and PGN (10 mg/kg, i.p.) were administered from 8 to 28 days after the STZ treatment. The measurements of body weight (A) and blood glucose levels (B) were performed before starting (0 days), and 7, 14, 21, and 28 days after the STZ treatment. The data are represented as the mean ± SD (n = 12). *P < .05, **P < .01 vs Non‐DM + NS group by two‐way repeated measures ANOVA followed by Dunn's comparison test. STZ, streptozotocin; EDR, edaravone; PGN, pregabalin; DM, diabetic mellitus; NS, normal saline; DN, diabetic neuropathy

The changes in body weight and blood glucose levels were measured to evaluate any body weight loss and development of hyperglycemia. Figure 2 exhibits time‐dependent changes in the body weight and blood glucose levels after STZ administration in mouse model of DN. The diabetes mellitus + NS (DM + NS) group showed a significant decrease in the body weight (*P* < .05, 21.83 ± 1.12 g) and an increase in the blood glucose levels (*P* < .01, 507.00 ± 93.69 mg/dL) at 7 day compared with the Non‐DM + NS group (body weight, 22.75 ± 0.45 g; blood glucose, 200.00 ± 23.12 mg/dL). However, the body weight and blood glucose levels in the treatment groups DM + SMTP‐44D (30 mg/kg), DM + EDR (10 mg/kg), and DM + PGN (10 mg/kg) remained unchanged compared with those in the DM + NS group.

### 3.2 Changes in the mechanical threshold in response to SMTP‐44D administration in STZ‐induced diabetic mice

**Figure 3:**
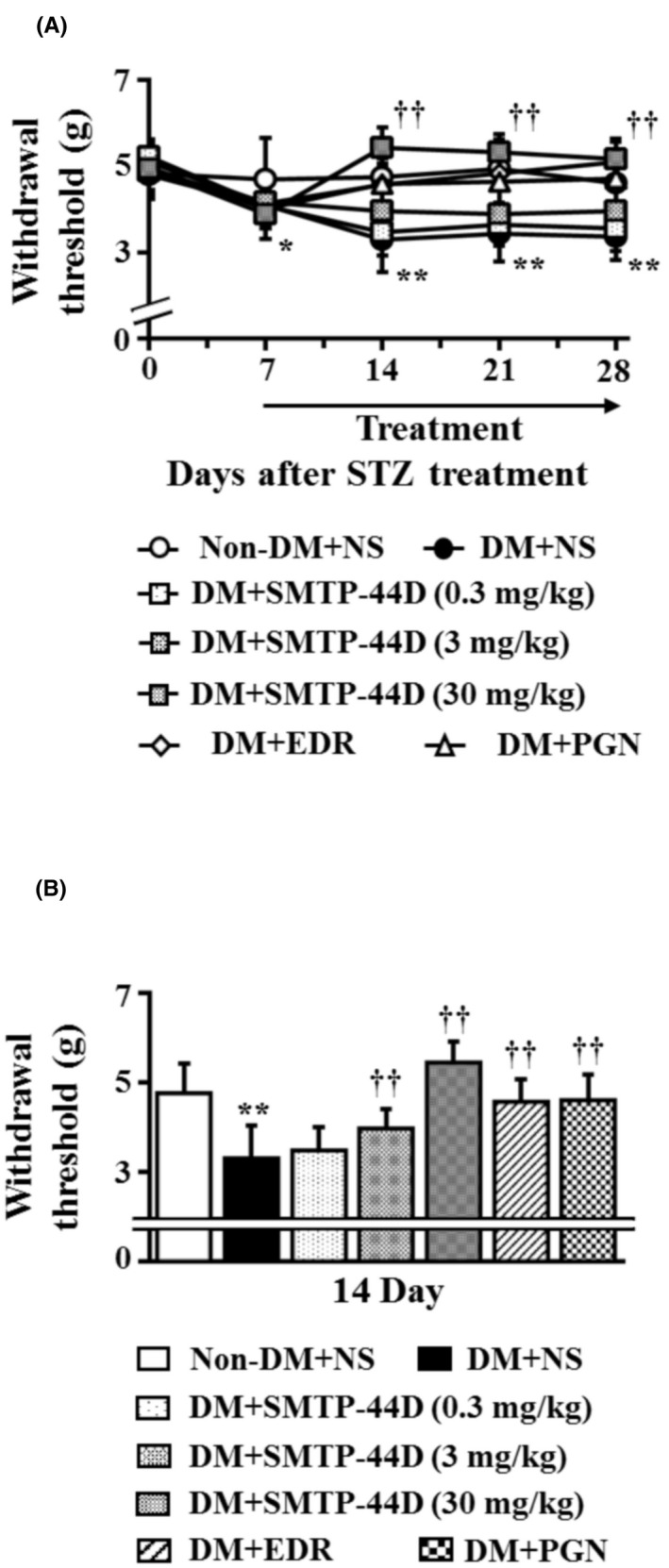
Time‐dependent and dose‐dependent changes in mechanical thresholds by the von Frey test in mouse model of DN. (A) shows the changes in mechanical thresholds from 0 to 28 days after STZ treatment, and (B) shows the changes in mechanical thresholds at 14 day. SMTP‐44D (0.3, 3, and 30 mg/kg, intraperitoneally [i.p.]), EDR (10 mg/kg, i.p.), and PGN (10 mg/kg, i.p.) were administered from 8 to 28 days after the STZ treatment. The measurements of (A) and (B) were performed before starting (0 days), and 7, 14, 21, and 28 days after the STZ treatment. The data are represented as the mean ± SD (n = 12). *P < .05, **P < .01 vs Non‐DM + NS group by two‐way repeated measures ANOVA followed by Dunn's comparison test; ^††^ *P* < .01 vs DM + NS group by one‐way ANOVA followed by Bonferroni test. STZ, streptozotocin; EDR, edaravone; PGN, pregabalin; DM, diabetic mellitus; NS, normal saline; DN, diabetic neuropathy

The mechanical thresholds were measured to evaluate allodynia. Figure 3 shows the time‐dependent changes after the STZ administration and dose‐dependent effect of SMTP‐44D as assessed by measuring the mechanical thresholds using the von Frey test. Mechanical thresholds were significantly decreased in the DM + NS group (*P* < .05, 4.11 ± 0.56 g) at 7 day compared with that in the Non‐DM + NS group (4.70 ± 0.96 g). The treatment groups, DM + SMTP‐44D (30 mg/kg), DM + EDR (10 mg/kg), and DM + PGN (10 mg/kg) showed a significant improvement in the mechanical thresholds (*P* < .01, 5.43 ± 0.47 g; *P* < .01, 4.56 ± 0.50 g; and *P* < .01, 4.59 ± 0.59 g, respectively) at 14 day compared with the DM + NS group (3.29 ± 0.74 g). The SMTP‐44D group (0.3 and 3 mg/kg) exhibited a dose‐dependent improvement in mechanical thresholds compared with the DM + NS group.

### 3.3 Changes in the thermal thresholds in response to SMTP‐44D administration in STZ‐induced diabetic mice

**Figure 4:**
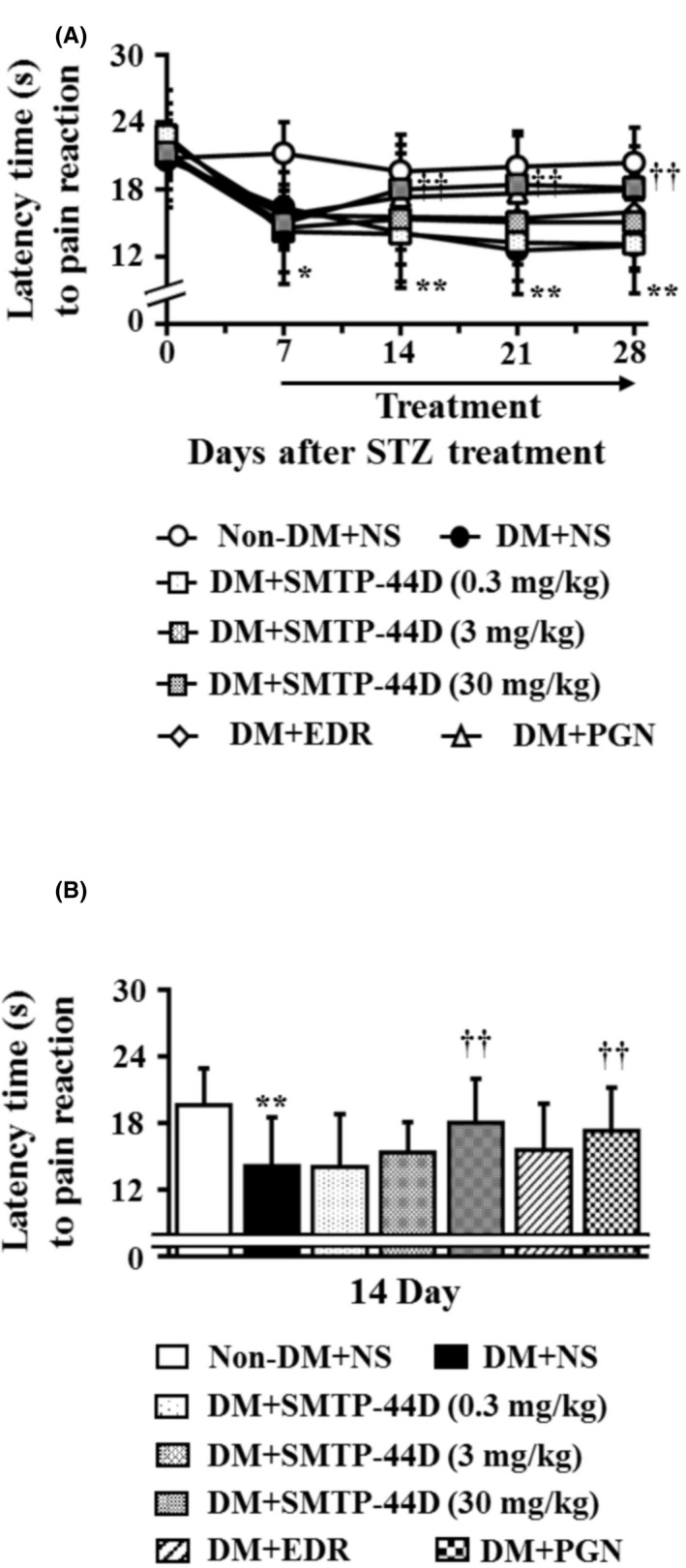
Time‐dependent and dose‐dependent changes in thermal thresholds by the hot plate test in mouse model of DN. (A) shows the changes in thermal thresholds from 0 to 28 days after the STZ treatment, and (B) shows the changes in thermal thresholds at 14 day. SMTP‐44D (0.3, 3, and 30 mg/kg, intraperitoneally [i.p.]), EDR (10 mg/kg, i.p.), and PGN (10 mg/kg, i.p.) were administered from 8 to 28 days after the STZ treatment. The measurements of (A) and (B) were performed before starting (0 days), and 7, 14, 21, and 28 days after the STZ treatment. The data are represented as the mean ± SD (n = 12). *P < .05, **P < .01 vs Non‐DM + NS group by two‐way repeated measures ANOVA followed by Dunn's comparison test; ^††^ *P* < .01 vs DM + NS group by one‐way ANOVA followed by Bonferroni test. STZ, streptozotocin; EDR, edaravone; PGN, pregabalin; DM, diabetic mellitus; NS, normal saline; DN, diabetic neuropathy; ANOVA, analysis of variance

The thermal thresholds were measured to evaluate hyperalgesia. Figure 4 shows the time‐dependent changes after STZ administration and dose‐dependent effect of SMTP‐44D on the thermal thresholds using the hot plate test. The DM + NS group showed a significant decrease in the thermal thresholds (*P* < .05, 16.42 ± 3.01 seconds) at 7 day compared with the Non‐DM + NS group (21.19 ± 2.77 seconds). The DM + SMTP‐44D (30 mg/kg) and DM + PGN (10 mg/kg) groups showed a significant improvement in the thermal thresholds (*P* < .01, 18.00 ± 3.97 seconds; and *P* < .05, 17.31 ± 3.93 seconds, respectively) at 14 day compared with the DM + NS group (14.14 ± 4.38 seconds). Furthermore, the DM + EDR (10 mg/kg) group showed a significant improvement in the thermal thresholds (*P* < .05, 15.97 ± 2.86 seconds) at 28 day compared with the DM + NS group (12.97 ± 4.28 seconds). The SMTP‐44D (0.3 and 3 mg/kg) group exhibited a dose‐dependent improvement in the thermal thresholds compared with the DM + NS group.

### 3.4 Changes in the blood flow in the bilateral hind paw skin in response to SMTP‐44D administration in streptozotocin‐induced diabetic mice

**Figure 5:**
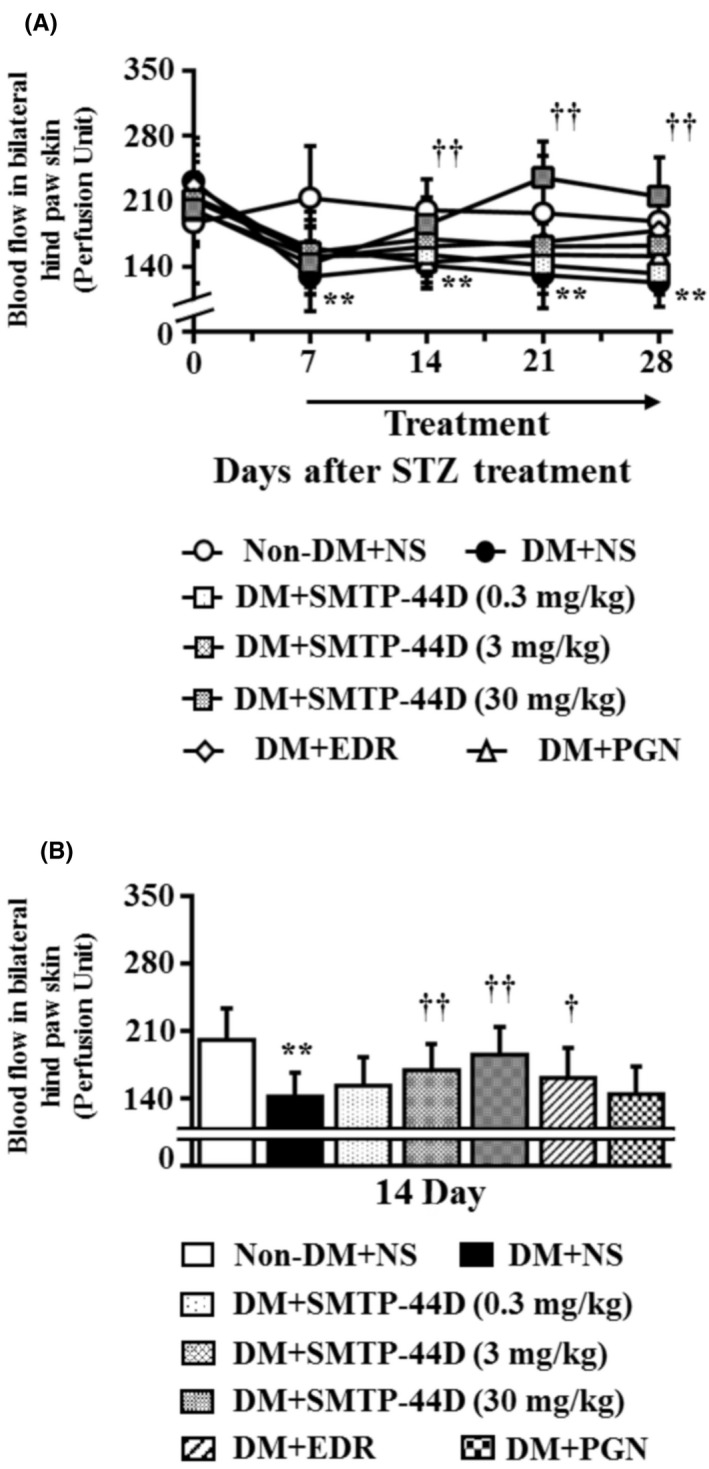
Time‐dependent and dose‐dependent changes in the blood flow in bilateral hind paw in the mouse model of DN. (A) shows the changes in the blood flow in the bilateral hind paw from 0 to 28 days after the STZ treatment, and (B) shows the changes in the blood flow in the bilateral hind paw at 14 day. SMTP‐44D (0.3, 3, and 30 mg/kg, intraperitoneally [i.p.]), EDR (10 mg/kg, i.p.), and PGN (10 mg/kg, i.p.) were administered from 8 to 28 days after the STZ treatment. The measurements of (A) and (B) were performed before starting (0 days), and 7, 14, 21 and 28 days after the STZ treatment. The data are represented as the mean ± SD (n = 12). **P < .01 vs Non‐DM + NS group by two‐way repeated measures ANOVA followed by Dunn's comparison test; ^†^ *P* < .05, ^††^ *P* < .01 vs DM + NS group by one‐way ANOVA followed by Bonferroni test. STZ, streptozotocin; EDR, edaravone; PGN, pregabalin; DM, diabetic mellitus; NS, normal saline; DN, diabetic neuropathy; ANOVA, analysis of variance

The blood flow in the bilateral hind paw skin was measured to determine any decrease in the blood flow. Figure 5 shows the time‐dependent changes after the STZ administration, and the dose‐dependent effects of SMTP‐44D on the blood flow in the bilateral hind paw skin. The DM + NS group showed a significant decrease in the blood flow in the bilateral hind paw skin (*P* < .01, 128.85 ± 36.78 perfusion unit [PU]) at 7 day compared with the Non‐DM + NS group (213.08 ± 55.76 PU). The DM + SMTP‐44D (30 mg/kg) and DM + EDR (10 mg/kg) groups showed a significant improvement in the blood flow in the bilateral hind paw skin (*P* < .01, 184.95 ± 29.18 PU; and *P* < .05, 161.35 ± 31.48 PU, respectively) at 14 day compared with the DM + NS group (141.66 ± 24.92 PU). Furthermore, the DM + PGN (10 mg/kg) group showed a significant improvement in the blood flow in the bilateral hind paw skin (*P* < .01, 151.17 ± 27.71 PU) at 28 day compared with the DM + NS group (122.50 ± 25.34 PU). The SMTP‐44D (0.3 and 3 mg/kg) group showed a dose‐dependent improvement in the blood flow in the bilateral hind paw skin compared with the DM + NS group.

### 3.5 Changes in the blood flow and conduction velocity in the sciatic nerve in response to SMTP‐44D administration in STZ‐induced diabetic mice

**Figure 6:**
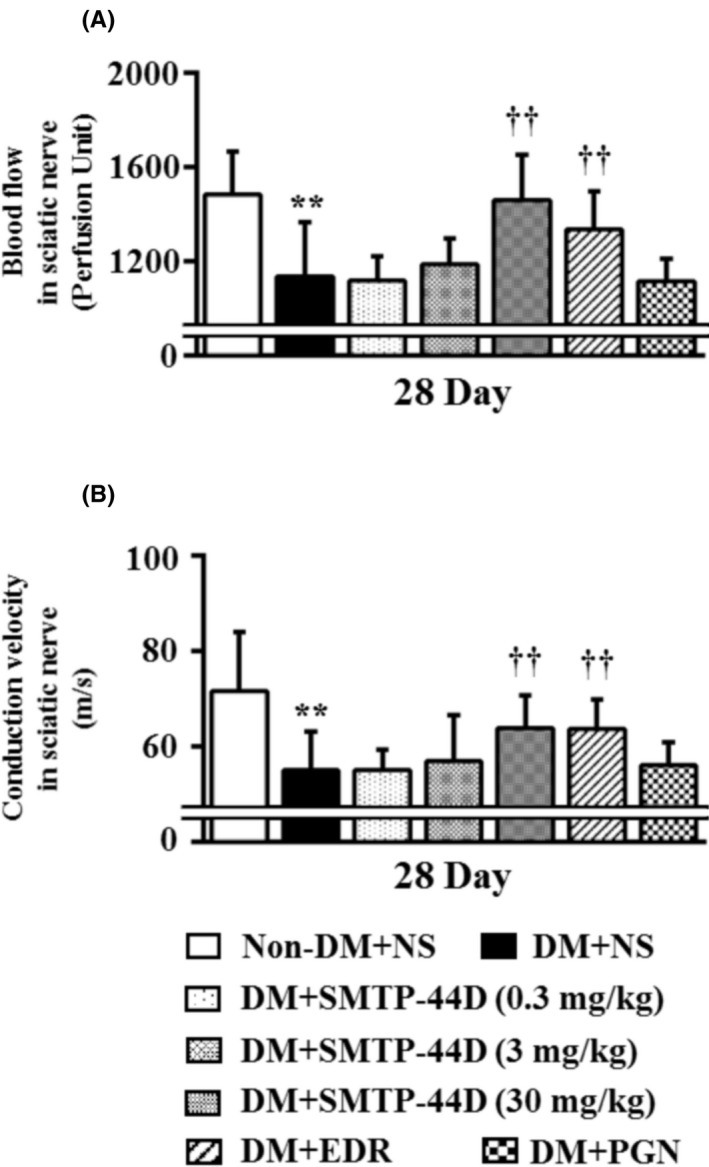
Dose‐dependent changes in the blood flow (A) and conduction velocity (B) in the sciatic nerve in the mouse model of DN. SMTP‐44D (0.3, 3, and 30 mg/kg, intraperitoneally [i.p.]), EDR (10 mg/kg, i.p.), and PGN (10 mg/kg, i.p.) were administered from 8 to 28 days after the STZ treatment. The measurements of (A) and (B) were performed at 28 day after the STZ treatment. The data are represented as the mean ± SD (n = 12). **P < .01 vs Non‐DM + NS group; ^††^ *P* < .01 vs DM + NS group by one‐way ANOVA followed by Bonferroni test. STZ, streptozotocin; EDR, edaravone; PGN, pregabalin; DM, diabetic mellitus; NS, normal saline; DN, diabetic neuropathy; ANOVA, analysis of variance

Blood flow and conduction velocity in the sciatic nerve were measured to determine any decrease in blood flow and conduction velocity. Figure 6 shows a dose‐dependent effect of SMTP‐44D on the blood flow and conduction velocity in the sciatic nerve at 28 day. The DM + NS group showed a significant decrease in the blood flow and conduction velocity (blood flow, *P* < .01, 1133.24 ± 231.30 PU; and conduction velocity, *P* < .01, 54.91 ± 8.22 m/s) compared with the Non‐DM + NS group (blood flow, 1481.92 ± 183.61 PU; and conduction velocity, 71.64 ± 12.36 m/s). The treatment groups DM + SMTP‐44D (30 mg/kg) and DM + EDR (10 mg/kg) showed a significant improvement in the blood flow and conduction velocity (*P* < .01, 1460.98 ± 190.49 PU; *P* < .01, 1336.07 ± 161.64 PU and *P* < .01, 63.79 ± 6.88 m/s; and *P* < .01, 63.56 ± 6.23 m/s, respectively) compared with the DM + NS group. However, the blood flow and conduction velocity in the DM + PGN (10 mg/kg) group remained unchanged compared with that in the DM + NS group. The SMTP‐44D group (0.3 and 3 mg/kg) showed dose‐dependent improvement in the blood flow and conduction velocity compared with the DM + NS group.

### 3.6 Changes in the myelin thickness and g‐ratio in response to SMTP‐44D administration in streptozotocin‐induced diabetic mice

**Figure 7:**
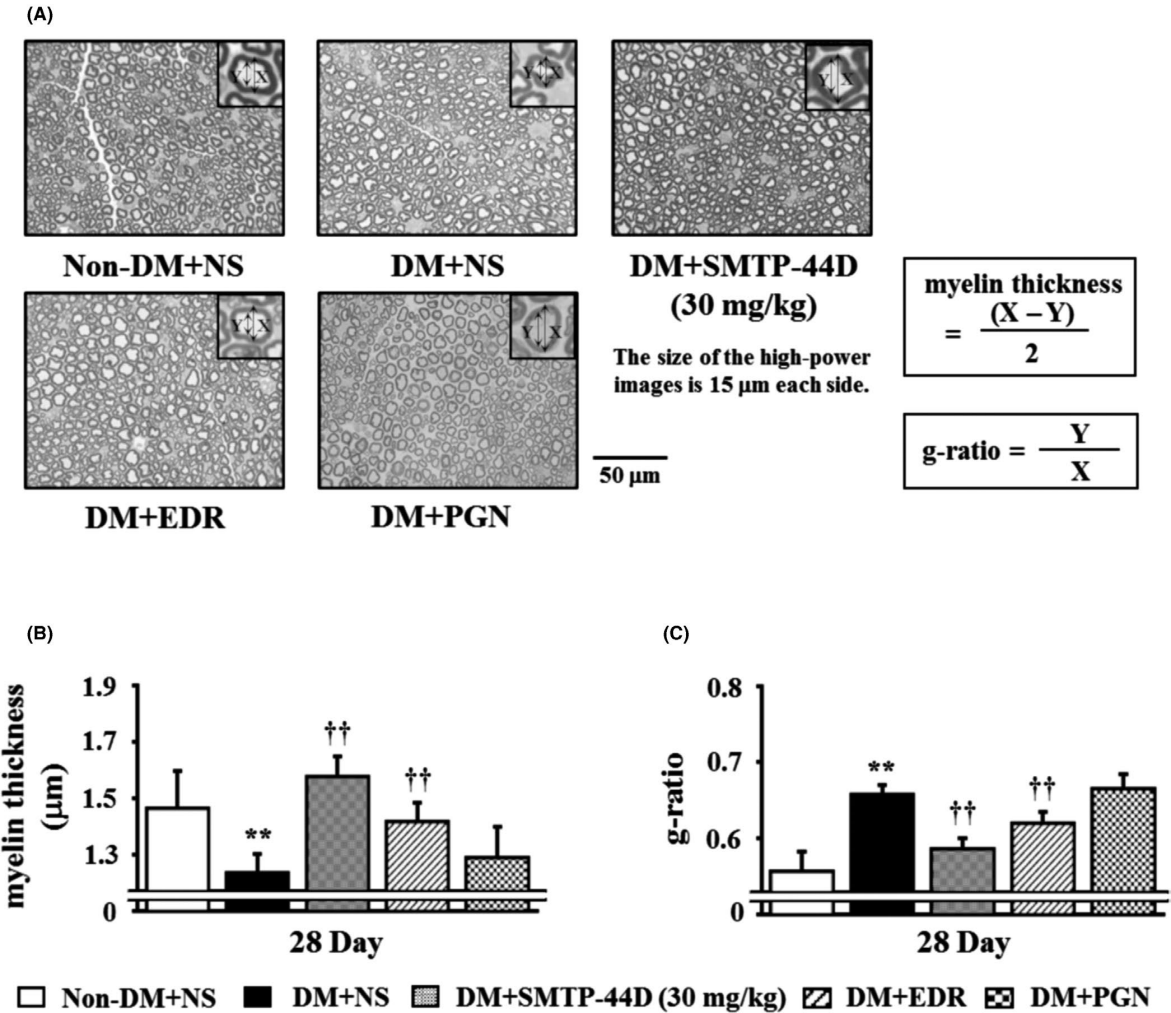
Evaluation of the myelin thickness (B) and g‐ratio (C) in the sciatic nerve in the mouse model of DN. The representative images (A) of toluidine blue stained transverse semi‐thin sections of the sciatic nerve at 400‐fold magnification are shown. X, myelinated fiber diameter (axon + myelin); Y, axon diameter. SMTP‐44D (30 mg/kg, intraperitoneally [i.p.]), EDR (10 mg/kg, i.p.), and PGN (10 mg/kg, i.p.) were administered from 8 to 28 days after the STZ treatment. The evaluation of (B) and (C) were performed at 28 day after the STZ treatment. The data are represented as the mean ± SD (n = 12). **P < .01 vs Non‐DM + NS group; ^††^ *P* < .01 vs DM + NS group by one‐way ANOVA followed by Bonferroni test. STZ, streptozotocin; EDR, edaravone; PGN, pregabalin; DM, diabetic mellitus; NS, normal saline; DN, diabetic neuropathy; ANOVA, analysis of variance

The myelin thickness and g‐ratio were measured to determine any neurological degeneration. Figure 7 shows the effects of SMTP‐44D administration on the myelin thickness and g‐ratio in the sciatic nerve at 28 day. The DM + NS group showed a significant decrease in the myelin thickness (*P* < .01, 1.236 ± 0.067 μm) and a significant increase in the g‐ratio (*P* < .01, 0.657 ± 0.013) compared with the Non‐DM + NS group (myelin thickness, 1.465 ± 0.134 μm; and g‐ratio, 0.556 ± 0.027). The DM + SMTP‐44D (30 mg/kg) and DM + EDR (10 mg/kg) groups showed a significantly improved myelin thickness (*P* < .01, 1.578 ± 0.072 μm; and *P* < .01, 1.418 ± 0.068 μm) and an increased g‐ratio (*P* < .01, 0.585 ± 0.015; and *P* < .01, 0.619 ± 0.016) compared with the DM + NS group. However, myelin thickness and g‐ratio in the DM + PGN (10 mg/kg) group remained unchanged compared with those in the DM + NS group.

### 3.7 Changes in the levels of oxidative stress and inflammatory cytokines in response to SMTP‐44D administration in streptozotocin‐induced diabetic mice

**Figure 8:**
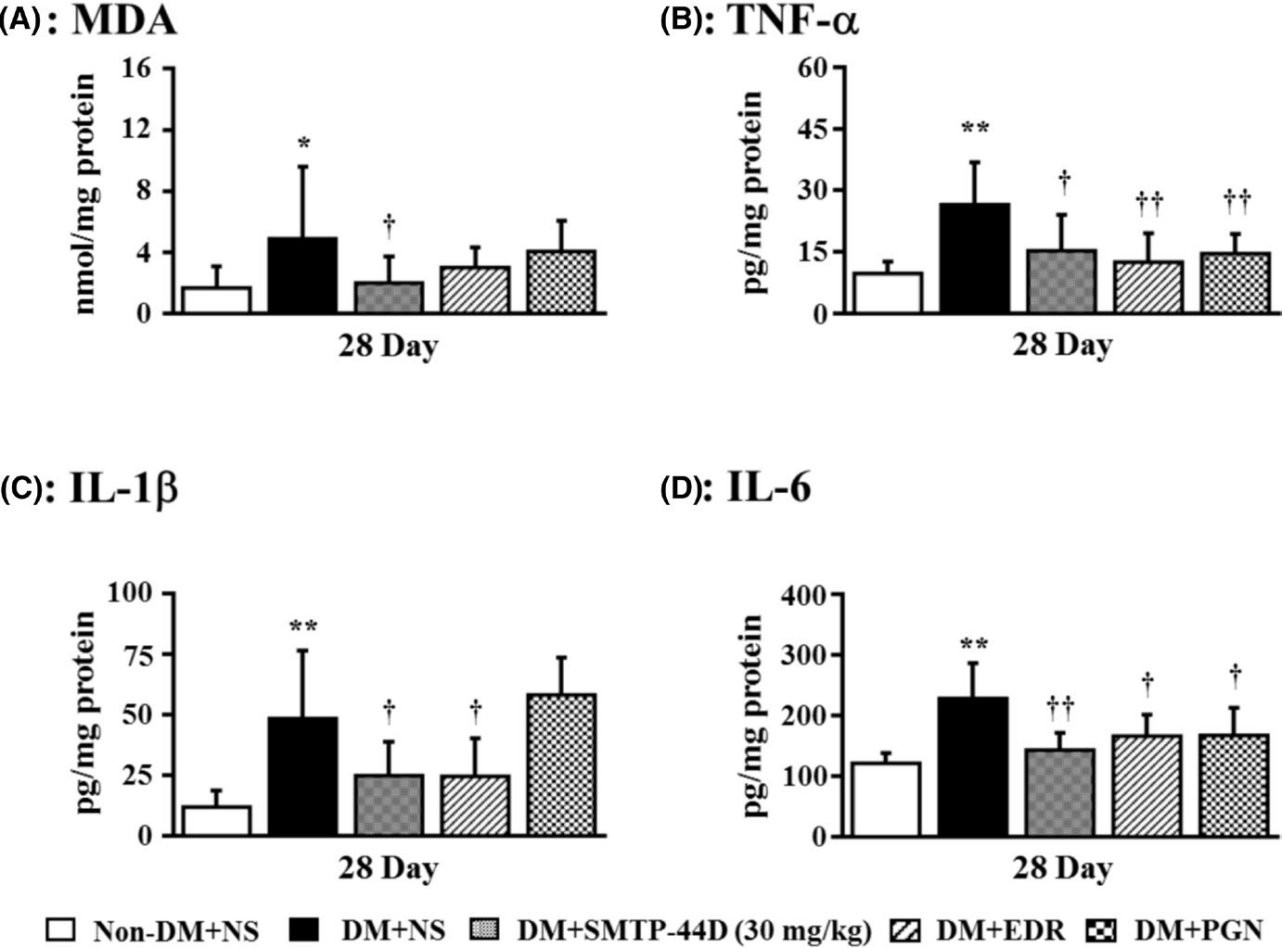
Effects of oxidative stress on the levels of MDA (A) and inflammatory cytokines TNF‐α (B), IL‐1β (C), and IL‐6 (D) in the mouse model of DN. SMTP‐44D (30 mg/kg, intraperitoneally [i.p.]), EDR (10 mg/kg, i.p.), and PGN (10 mg/kg, i.p.) were administered from 8 to 28 days after the STZ treatment. The effects on (A) to (D) were determined at 28 day after the STZ treatment. The data are represented as the mean ± SD (n = 12). *P < .05, **P < .01 vs Non‐DM + NS group; ^††^ *P* < .01 vs DM + NS group by Kruskal‐Wallis ANOVA followed by Dunn's comparison test. STZ, streptozotocin; EDR, edaravone; PGN, pregabalin; DM, diabetic mellitus; NS, normal saline; DN, diabetic neuropathy; ANOVA, analysis of variance; MDA, malondialdehyde

The levels of oxidative stress and inflammatory cytokines were measured to evaluate the exacerbation of inflammation. Figure 8 summarizes the effects of SMTP‐44D administration on MDA levels as assessed by TBARS assay and the levels of TNF‐α, IL‐1β, and IL‐6 as assessed by ELISA at 28 day. In the DM + NS group, the levels of MDA (*P* < .05, 4.86 ± 4.74 nmol/mg protein), TNF‐α (*P* < .01, 26.35 ± 10.41 pg/mg protein), IL‐1β (*P* < .01, 48.42 ± 28.13 pg/mg protein), and IL‐6 (*P* < .01, 228.39 ± 58.29 pg/mg protein) were significantly increased as compared to that in the Non‐DM + NS group (1.65 ± 1.42 nmol/mg protein; 9.68 ± 2.93 pg/mg protein; 11.89 ± 6.97 pg/mg protein; and 121.43 ± 17.46 pg/mg protein, respectively). The DM + SMTP‐44D (30 mg/kg) treatment group showed a significant decrease in the levels of MDA (*P* < .05, 1.98 ± 1.76 nmol/mg protein), TNF‐α (*P* < .05, 15.22 ± 8.70 pg/mg protein), IL‐1β (*P* < .05, 24.90 ± 14.04 pg/mg protein), and IL‐6 (*P* < .01, 143.41 ± 28.00 pg/mg protein) compared with the DM + NS group. Furthermore, the DM + EDR (10 mg/kg) group showed a significant decrease in the upregulated levels of TNF‐α (*P* < .01, 12.45 ± 7.12 pg/mg protein), IL‐1β (*P* < .05, 24.54 ± 15.66 pg/mg protein), and IL‐6 (*P* < .05, 165.83 ± 35.37 pg/mg protein) compared with the DM + NS group. The levels of MDA in the DM + EDR (10 mg/kg) group showed a tendency to decrease in comparison to that in the DM + NS group, but this was not statistically significant. In addition, the DM + PGN (10 mg/kg) treatment group showed significantly improved levels of TNF‐α (*P* < .01, 14.53 ± 4.87 pg/mg protein) and IL‐6 (*P* < .05, 166.60 ± 47.02 pg/mg protein) in comparison with that in the DM + NS group, while the levels of MDA and IL‐1β in the DM + PGN (10 mg/kg) group remained unchanged in a comparison with that in the DM + NS group.

## 4 DISCUSSION

The present study shows that SMTP‐44D can dose‐dependently improve mechanical allodynia and thermal hyperalgesia, decrease the blood flow, and delay the conduction velocity without affecting the body weight and blood glucose levels.

The STZ‐induced mouse model is the most widely used experimental model of human diabetes[27, 28] and is characterized by sensory defects, such as mechanical allodynia and thermal hyperalgesia, a decrease in blood flow, a delay in conduction velocity, myelin degeneration, and an induction of oxidative stress with increased inflammatory cytokine response, which are similar to those observed in the human disease. However, there has been a lack of consistency regarding the methods that have been used for the evaluation of DN; thus, it is necessary to establish an evaluation method against DN. In our preliminary study, we determined the useful experimental method against DN. It was reported that STZ‐induced diabetic mice developed mechanical allodynia and thermal hyperalgesia, the main indictors of pain‐related behavior at 1‐14 and 1‐4 weeks, respectively,[29, 30] and developed hyperalgesia after 6 weeks.[31, 32] Therefore, to assess both allodynia and hyperalgesia, we examined the mice for up to 4 weeks after the STZ treatment. Mechanical allodynia and thermal hyperalgesia develop in diabetic rodent models owing to multiple pathogenic mechanisms, such as oxidative stress and inflammation.[33] Several human and animal studies have demonstrated that hyperglycemia‐associated damages to unmyelinated C‐fibers, small myelinated Aδ‐fibers, and large myelinated Aβ‐fibers contributed to the development of DN.[34] The enhanced firing and sensitization of these injured fibers due to oxidative stress and inflammation lead to mechanical allodynia and thermal hyperalgesia in DN. The decrease in the blood flow and delay in conduction velocity are the indicators of DN pathogenesis and are caused by enhanced oxidative stress resulting from an imbalance between reactive oxygen species (ROS) production and neutralization.[35] ROS are pro‐oxidant factors in diabetes, and tissue damage mediated by lipid peroxides has been observed in type 1 and type 2 diabetes.[36] In recent studies, major diabetic complications have been associated with inflammation.[37] Particularly, neuropoietic cytokines, such as TNF‐α, IL‐1β, and IL‐6, play a vital role in systemic inflammation and propagation of the acute phase reaction.[38] The induction of inflammation has been related to microvascular and neural tissue damage in both the human diabetes and the rodent models of diabetes.[39, 40] Moreover, neural tissue damage, such as myelin degeneration, is one of the main symptoms of DN. Structural changes, including axon atrophy and loss of myelinated nerve fibers, are characteristics of DN. Neural tissue damage due to oxidative stress and inflammation can lead to a decrease in blood flow and delay in conduction velocity in DN.

We detected neuropathy associated with the STZ‐induced mouse model by evaluating mechanical allodynia, thermal hyperalgesia, blood flow, and conduction velocity (Figures 3, 4, 5, 6). These results were similar to other reports.[8, 41] Thus, this evaluation method was found to be suitable for evaluating the pathological conditions and therapeutic targets of DN.

We found that SMTP‐44D had anti‐allodynic and anti‐hyperalgesic effects in this study. To understand these effects, we chose PGN as a standard drug.[42] EDR demonstrated antioxidant and anti‐inflammatory activities and was reported to have improved the DN in STZ‐induced rats.[8] Thus, we chose EDR as SMTP‐44D had similar activities. EDR was the neuroprotective drug, and has been used to treat many acute ischemic stroke patients in Japan since 2001. The antioxidant effects of EDR are mediated by its potent free radical scavenging activity that inactivates the hydroxyl radicals (·OH) and inhibits ·OH‐dependent and ·OH‐independent lipid peroxidation,[43] it is used to evaluate antioxidant and anti‐inflammatory activities. The EDR dose was selected as reported previously.[44, 45, 46] PGN is the first line agent for the treatment of painful DN. It binds to the α_2_δ subunit of the voltage dependent calcium channel in the central nervous system indicating an anti‐hyperalgesic and anti‐allodynic effect specific for its action at the CaVα_2_δ‐1 subunit.[47] Strong binding at this channel reduces calcium inflow at the nerve terminals that reduce the release of several neurotransmitters including substance P, glutamate, and norepinpherine.[48, 49] In basic experiments, it is used to evaluate anti‐allodynic and anti‐hyperalgesic effects. The PGN dose was selected as reported previously.[42, 50] In this study, we confirmed the antioxidant and anti‐inflammatory activities of EDR and the anti‐allodynic and anti‐hyperalgesic effects of PGN (Figures 3, 4, 5, 6). Thus, the antioxidant and anti‐inflammatory activities of EDR, and the anti‐allodynic and anti‐hyperalgesic effects of PGN were useful as controls for DN in the basic experiments. However, in clinical practice, it was reported that EDR was a risk factor for acute kidney and liver injuries.[51, 52] PGN has a risk factor of drowsiness and dizziness. In addition, because PGN is just the calcium channel blocker and has no direct antioxidant and anti‐inflammatory activities, it may not repair nerve damage. Thus, EDR and PGN are inadequate as therapeutic agents. Therefore, SMTP‐44D ameliorates the pathological condition associated with DN through its antioxidant and anti‐inflammatory activities, and thus, has therapeutic potential against DN. However, no toxicity data is available in this study regarding SMTP‐44D. Owing to our aim of finding the therapeutic potential against the DN of SMTP‐44D, we did not evaluate its toxicity.

The therapeutic effects of SMTP‐44D were evaluated using the mouse model of DN generated in this study. SMTP‐44D showed improvement of mechanical allodynia and thermal hyperalgesia symptoms of DN. This is suggested that these symptoms improve accompanying decreased levels of inflammatory factors. Additionally, EDR also has potent antioxidant and anti‐inflammatory properties, suggesting that it may yield similar results compared to SMTP‐44D. Notably, oxidative stress and inflammation seem to play important roles in the progression of DN.[14, 50, 53] Furthermore, it is considered that these effects recover the decrease of blood flow and delay of conduction velocity, and improve the symptoms of DN. In addition, the microvasculature dysfunction caused by hypoxic neuropathy accompanies demyelination. The axons with a severe loss of myelination in the peripheral nerves and microvascular damage either precede or parallel the impairment of nerve function as DN progresses.[54] Therefore, it is suggested that the decrease in blood flow, delay of conduction velocity, and myelin degeneration in the sciatic nerve are closely related. Furthermore, a decrease in blood flow in the bilateral hind paw from 7 day is also considered to correlate with the delay of conduction velocity and neurological degeneration. Hence, the improvement of myelin degeneration in sciatic nerve due to SMTP‐44D is also considered to be a factor that improved the symptoms of DN.

SMTP44D is superior to EDR and PGN in improving the symptoms to normal levels. Interestingly, SMTP‐44D has been found to have therapeutic potential against DN because it repaired the damage of myelinated sciatic nerve and improved the neural function. Thus, the use of SMTP‐44D has important clinical implications.

In spite of the intensive blood glucose control, the prevention of the progression of DN has not been successful.[10, 55] This may be due to metabolic and enzymatic changes, and a nerve damage that occur owing to persistent hyperglycemia before exercise or use of anti‐diabetes agent is started after the diagnosis of diabetes. When these changes abnormally activate the pathways of glucose metabolism, such as protein kinase C pathway and polyol pathway, it is possible to cause the dysregulation of cytokine control by the persistent hyperglycemia.[40] This could progress into DN through the activation of NF‐κB that can exacerbate the inflammatory mediators, such as TNF‐α, IL‐1β, and IL‐6 thorough persistent activation of the oxidative stress pathway regardless of the blood glucose control.[56] The exacerbations of these mediators may also be involved in microvascular damage. SMTP‐44D‐treated animals showed an improvement in the blood flow, conduction velocity, and myelin degeneration without significantly changing the body weight and blood glucose compared with the DM + NS group. This result suggests that antioxidant and anti‐inflammatory activities may be involved in suppressing the release of these mediators. In addition, SMTP‐44D may improve the symptoms of DN through its yet unknown effects. In the future, it is necessary to research in detail the mechanism of action of SMTP‐44D against DN.

In conclusion, SMTP‐44D improves the neural function, mechanical allodynia, and thermal hyperalgesia associated with DN through its antioxidant and anti‐inflammatory activities. Thus, SMTP‐44D has therapeutic potential against DN.

## AUTHOR CONTRIBUTIONS

Shinouchi, Shibata, Hashimoto and Nobe participated in research design. Shinouchi, Shibata, and Jono conducted experiments. Hasumi contributed new reagents. Shinouchi and Jono performed data analysis. Shinouchi, Shibata, Hasumi and Nobe wrote or contributed to the writing of the manuscript.

### OPEN RESEARCH BADGE

This article has earned Open Data, Open Materials and Preregistered Research Design badges. Data, materials and the preregistered design and analysis plan are available in the article.

## Data Availability

We share the data and other artifacts supporting the results in the paper by archiving it in an appropriate public repository.
